# Fostering Elementary School Students’ Self-Regulation Skills in Reading Comprehension: Effects on Text Comprehension, Strategy Use, and Self-Efficacy

**DOI:** 10.3390/bs15020101

**Published:** 2025-01-21

**Authors:** Irini Dermitzaki

**Affiliations:** Department of Early Childhood Education, University of Thessaly, 38221 Volos, Greece; idermitzaki@uth.gr; Tel.: +30-24210-74790

**Keywords:** reading comprehension, self-regulated learning, metacognitive skills, self-efficacy in reading comprehension, intervention

## Abstract

It has been maintained that active strategic reading is beneficial for students’ reading comprehension (RC) and has cognitive, motivational, and behavioral benefits for students. The aim of the present study was to implement and evaluate the effectiveness of a self-regulation skills training program with a focus on RC for elementary school students. One hundred and nine students (60 girls) from 5th and 6th grade were randomly assigned to an experimental (EG) and a control group (CG). The EG (*n* = 54) took an intervention program aiming at fostering students’ cognitive and metacognitive strategies to control and regulate the RC process. Before and after the intervention, students’ performance in RC, reported use of strategies for RC, and self-efficacy regarding RC were assessed. The analyses of the data showed that, after the intervention, the EG’s reported strategy use and RC performance were significantly improved in comparison to the CG. Students’ self-efficacy regarding RC did not improve significantly in either group, indicating that in order to increase self-efficacy for RC longer-term interventions might be needed. A follow-up three months after the intervention attested to the maintenance of the core elements of the training program. The findings of the study are discussed within the socio-cognitive approach of self-regulated learning.

## 1. Introduction

Reading comprehension is a key skill for students in order to be academically and socially proficient. It has been proposed that reading comprehension is the active extraction and construction of meaning from text (Kintsch, 2009). A skilled reader is able to process a text at various levels, superficially and deeply, in order to build understanding. To interact effectively with the text and to construct meaning, a student has to activate, among others, previous knowledge, complex mental and affective processes, but also strategies and skills to control and regulate the reading process (Y.-S. G. Kim, 2017; Kintsch, 2009; Pressley, 2002; Smith et al., 2008; Snow, 2010). Hence, it has been claimed that learning from text demands readers who are strategically engaged in the construction of meaning (Pressley & Gaskins, 2006).

Reading strategies, which are deliberate, planful activities undertaken by active learners to regulate aspects of the reading process, have been found to facilitate reading comprehension (RC) (Connor et al., 2016; Mägi et al., 2016; Mokhtari & Reichard, 2002; Schunk & Zimmerman, 2007). The view of an active, self-regulated reader who masters and uses appropriately multiple skills and strategies to build understanding is aligned with contemporary approaches of self-regulated learning and reading (Azevedo et al., 2004; Efklides, 2011; Gourgey, 2002; Mason, 2013; Skibbe et al., 2019; Zimmerman & Schunk, 2011). Self-regulated learning (SRL) refers to the degree to which students are metacognitively, motivationally, and behaviorally active participants in their own learning processes (Zimmerman, 2013). It has been defined that self-regulated learners deliberately set their own goals for learning, monitor and control their thinking, use strategies that ensure learning outcomes, and reflect on their learning behaviors vis-à-vis their goals and outcomes (Efklides & Schwartz, 2024). Research on reading strategy instruction suggests that SRL might be a powerful framework to optimize effects on RC (Connor et al., 2016; Mägi et al., 2016; Mason, 2013; Schunk & Zimmerman, 2007; Spruce & Bol, 2015).

In addition, it has been claimed that active reading is motivated reading, and good readers understand that they can derive meaning from text by expending the effort to use effective text processing strategies (Pressley & Gaskins, 2006). An important construct with motivational power is students’ academic self-efficacy that is significantly associated with strategic learning at different ages (Berger & Karabenick, 2011; Dermitzaki & Papakosma, 2020; D.-H. Kim et al., 2015). Academic self-efficacy can predict students’ use of learning strategies in various school subjects such as in language and maths, especially when self-efficacy is measured with reference to these subjects (Chatzistamatiou et al., 2015; Cleary & Kitsantas, 2017; D.-H. Kim et al., 2015). Following prominent researchers, we assert that active strategic reading has cognitive, motivational, and behavioral benefits for students and that teachers should be able to foster SRL skills in regular classrooms (Mokhtari & Reichard, 2002; Pressley & Gaskins, 2006; Trabasso & Bouchard, 2002). The literature has shown that training SRL skills is associated with improved academic performance and improved academic self-efficacy (Dignath & Büttner, 2008; Harding et al., 2019; Hessels-Schlatter et al., 2017; Schunk & Zimmerman, 2007). Teachers who use explicit instruction and modeling of SRL strategies have larger numbers of students who can use self-regulation to read for longer periods and respond to higher order thinking questions (Housand & Reis, 2008). However, respective evidence has also suggested that teachers usually do not explicitly teach strategies or create learning environments that promote SRL in students (Lawson et al., 2019; Spruce & Bol, 2015).

Moreover, most interventions intending to foster self-regulation in RC were implemented with students with special characteristics, such as difficulties in reading or special educational needs (e.g., Antoniou & Souvignier, 2007; Berkeley & Larsen, 2018; Mason, 2013), with at-risk students (e.g., Smith et al., 2008), and with highly achieving students (e.g., Housand & Reis, 2008; Sontag & Stoeger, 2015). Further, most of the interventions in regular classrooms with a focus on reading or RC have been applied on secondary school students (e.g., Butler et al., 2011; Lau, 2020) and on college students (e.g., Dörrenbächer & Perels, 2016; Salmerón et al., 2010). Instructional interventions addressed to typical elementary school classrooms in order to foster SRL skills in RC have been limited over the years (e.g., DeCorte et al., 2001; Souvinger & Mokhlesgerami, 2006; Spörer & Schünemann, 2014).

Therefore, further documentation on structured programs and practices is needed in order to better support both students and teachers to bring SRL to typical elementary schools. The aim of this study was to develop, implement, and evaluate a training program for upper elementary school students in order to enhance skilled strategy use in RC and related performance. Students’ self-efficacy in RC was also measured before and after the intervention as it is related to strategy use. The evidence from the present study could add to the respective literature, practices, and materials for educators in order to promote SRL in RC. Over the next pages, a literature review with a focus on an SRL framework in RC is presented, and the rationale and hypotheses of the study are outlined.

### 1.1. Reading Comprehension and SRL Skills

Current theoretical approaches on RC have embraced the contribution of active strategy use for the outcomes of the RC process. For example, in Kintsch’s (2009) model, it is maintained that RC involves conscious effort and the use of appropriate strategies on the behalf of the reader can, among other factors, contribute to meaning construction. He also claimed that different levels of text representation are related to different strategies employed for achieving comprehension. Furthermore, contemporary multicomponent models of RC endorse the contribution of strategies and skills to RC (e.g., Cromley & Azevedo, 2007; Y.-S. G. Kim, 2017). Snow (2010) has argued that in comprehension instruction for younger readers a focus on self-monitoring would be valuable to ensure that the process of reading remains focused on building mental representations and not just on reading the words.

Within the SRL framework, active employment of different groups of skills and strategies is considered as a core component of SRL (Dermitzaki et al., 2009; Efklides & Schwartz, 2024; Mason, 2013). Among the groups of strategies most frequently studied are (a) cognitive strategies, that is, strategies to rehearse, organize, and elaborate material, such as rereading, concept mapping, summarizing, (b) metacognitive strategies, like planning, self-monitoring, and self-evaluation of the learning process, and (c) strategies to regulate motivation and emotions, like self-motivation and managing anxiety (Dermitzaki et al., 2009; Efklides, 2011; Mason, 2013; Mokhtari & Reichard, 2002; Souvinger & Mokhlesgerami, 2006; Taboada et al., 2009). Research evidence supports that at around 11–12 years of age students’ metacognitive skills and individual ability to use SRL strategies to read and learn set in (Cobb, 2017; Housand & Reis, 2008; Kolić-Vehovec & Bajšanski, 2006; Veenman & Spaans, 2005).

It has been maintained that good readers know what strategies to use in order to achieve RC as well as when and where to use such strategies as they read (Paris & Oka, 1986; Pressley & Gaskins, 2006). Following Zimmerman’s three-phase model of SRL (Zimmerman, 2000), it has also been asserted that strategic action of good readers is present before, during, and after reading (Mason, 2013; Pressley & Gaskins, 2006). Before reading, a good reader previews, predicts, and plans his activities by deciding the steps of action and the means/strategies through which he will increase the possibilities to achieve the final goal (Pressley & Gaskins, 2006). Once actual reading begins, skilled readers are able to distinguish important information or to skip information that is not relevant to their reading goals, to predict what is coming up next, and to analyze and combine activities and information (Gourgey, 2002). Skilled readers while reading might also activate prior knowledge and appropriate strategies, generate questions, and pay attention to confusing points (Pressley & Hilden, 2006). Good readers are also highly interpretive and evaluative of the text during reading and they carry out a lot of monitoring of the reading and the comprehension processes. Over the reading process, they might need to adapt their goal or to adjust their strategies (Bråten et al., 2013; Horner & Shwery, 2002). Furthermore, successful readers persist in the face of reading difficulties and motivate themselves effectively throughout the activity. When good readers make it through a text once, they evaluate themselves to confirm that they understand and remember what they have read and they might deliberately reflect on the text (Horner & Shwery, 2002; Pressley & Hilden, 2006). These different phases of the reading act are reflected in models of intervention for students with difficulties or special needs in reading and writing, such as the “Think before reading, While reading, After reading” (TWA) (Mason, 2013) and the “Self-Regulated Strategy Development” (SRSD) models (Graham & Harris, 2018).

The associations between aspects of students’ SRL skills and cognitive attainments in reading and RC have been demonstrated in previous studies (Harding et al., 2019; Hessels-Schlatter et al., 2017; Mägi et al., 2016; Minguela et al., 2015; Pressley, 2002). Indeed, in a past study (Dermitzaki et al., 2008), the actual use of SRL strategies in RC was investigated in third graders via observation. High achievers in RC outperformed low achievers regarding their use of metacognitive strategies, such as planning, and regarding the use of deep processing strategies, such as choosing between main and trivial information. In another study, Minguela et al. (2015) highlighted the importance of self-regulated comprehension in 15–16-year-old students, especially the role of active online monitoring when reading for deep comprehension. Less-skilled readers approached the text more linearly and routinely, while skilled readers were more flexible. Self-regulated comprehension significantly predicted performance beyond readers’ comprehension skill. Focusing on RC in English as a second language (level 2), a meta-analysis (Vettori et al., 2023) pointed out the use of appropriate problem-solving strategies, such as rereading, reading carefully, and guessing the meaning of unfamiliar words and phrases, in English RC. More recently, it has been reported that 5th and 6th grade students with learning disabilities and comprehension difficulties reported more surface strategies and lower scores of adaptive strategies (deep, motivational, monitoring, and persistence) than their typical peers (Kampylafka et al., 2023). Such findings highlight the importance of enhancing students’ self-regulated strategic text comprehension.

Accordingly, training students to use a repertoire of comprehension strategies in a self-regulated fashion is reported to make a difference in the understanding of text and in respective performance (Pressley & Hilden, 2006). In an earlier meta-analysis of intervention studies, the effect size average of SRL interventions for reading performance was reported to be 0.44 for primary school (Dignath & Büttner, 2008). In a previous experimental study, a training program was implemented with a focus on teaching cognitive and metacognitive strategies that facilitate text comprehension (DeCorte et al., 2001). In this study, the experimental group outperformed the control group in terms of the strategy adoption and application during text reading. Whilst the experimental group also scored higher on the Reading Comprehension Test than the control group, the difference was not significant. In another experimental study, fifth graders were taught strategy knowledge along with skills of cognitive and motivational aspects of self-regulation (Souvinger & Mokhlesgerami, 2006). Three different strategy programs and one control condition were compared. All strategy-oriented programs proved to enhance reading competence, understanding of reading strategies, and competence for application of reading strategies. In another study, the effects of reciprocal teaching (RT) of reading strategies or RT and SRL on RC were examined (Schünemann et al., 2013). Evidence showed that, relative to RT students, students in the RT + SRL condition were better able to maintain training-induced performance gains. Moreover, it has been reported that students assigned to three self-regulation conditions outperformed RT students according to a standardized measure of RC (Spörer & Schünemann, 2014). Further, in another study, a significant impact of a teacher-led intervention on SRL and RC in 4th-grade students implemented during regular classroom instruction and homework was reported (Stoeger et al., 2014). Another intervention program for secondary school students was applied based on the instructional principles of SRL, and its effectiveness in enhancing RC and motivation (Lau, 2020). The findings of this study indicated that the students taught instructional materials from the intervention program had significantly better RC performance and prior knowledge than the control group. Finally, other studies indicated a reciprocal developmental relation between students’ reading comprehension and use of reading strategies (Connor et al., 2016; Muijselaar et al., 2017; Samuelstuen & Bråten, 2005).

### 1.2. Associations of Self-Efficacy in Reading with Students’ SRL Skills

According to Bandura’s (1997) social cognitive theory, self-efficacy and self-regulation are key processes that affect students’ learning and achievement. Self-efficacy reflects “people’s judgments of their capabilities to organize and execute courses of action required to attain designated types of performances” (Bandura, 1997, p. 391). Empirical evidence suggests that self-efficacy in a specific learning domain is a powerful motivational factor that predicts strategic learning in the respective domain, such as in text comprehension (Dermitzaki & Papakosma, 2020; Schunk & Zimmerman, 2007; Solheim, 2011; Walker, 2003), in learning English as a second language (Chen, 2022; D.-H. Kim et al., 2015), and in mathematics (Berger & Karabenick, 2011; Chatzistamatiou et al., 2015; Cleary & Kitsantas, 2017).

Further, it has been observed that interventions to enhance SRL skills may in turn positively affect students’ academic self-efficacy. It has been postulated that, after an intervention, the experience of deploying SRL strategies to successfully complete tasks could be conveyed to learners’ beliefs of their competence, thus positively affecting their self-efficacy (Bai & Guo, 2018; Chen, 2022). Students’ self-efficacy for reading is enhanced when they learn reading strategies and have opportunities for success in reading (Schunk & Zimmerman, 2007). For instance, in a recent study, after training students’ SRL skills it has been reported that, after three different instruction contexts regarding French learning, 11–12-year-old students’ reading self-efficacy was improved, and reading scores were the strongest predictor of reading self-efficacy (Graham et al., 2020). A few meta-analyses reported small to medium average effects of SRL training programs on students’ self-efficacy at various ages and in various learning domains. Specifically, the reported effect of self-assessment skills on students’ self-efficacy was 0.73 (Panadero et al., 2017), while at the tertiary education level, differential training effects of SRL interventions on students’ self-efficacy obtained an overall effect of *g* = 0.38 (Theobald, 2021). In addition, it was found that certain self-assessment components, such as self-monitoring, were significant moderators of the effects on self-efficacy (Panadero et al., 2017). In another meta-analysis regarding foreign language learning at level 2, SRL interventions influencing students’ self-efficacy obtained a small average effect *g* = 0.45 for both younger and university students (Chen, 2022).

However, evidence on the effects of SRL training programs on students’ self-efficacy with a focus on reading and RC is not always in agreement. There are studies that, although targeting increasing students’ self-efficacy via training SRL strategies, failed to report significant or direct effects of training on self-efficacy. Souvinger and Mokhlesgerami (2006) implemented an SRL strategy instruction program to foster RC in fifth graders. They reported that, within a pre-, post-, and retention-test design comprising development of reading comprehension and school-related self-efficacy, all strategy-oriented programs enhanced reading competence and competence for application of reading strategies, however, gains in self-efficacy did not differ from the control condition. Although the complete program revealed the most promising results according to cognitive variables, it did not lead to significant effects on motivational variables. In an intervention study on Chinese adolescents targeting improving RC, students of two out of the tree groups had significantly better RC performance, however, all groups did not demonstrate significant change in their self-efficacy (Lau, 2020). Another study aimed to investigate how training of university students in SRL strategies is related to improvements, among other factors, in self-efficacy in using those strategies and their effective use in academic learning tasks (Cerezo et al., 2019). No direct effects of training on self-efficacy were found, only indirect effects through knowledge of SRL strategies.

To sum up, it seems that there is a cyclical relationship between academic self-efficacy, strategy use, and students’ learning outcomes. Reader self-efficacy grows with comprehension skill, which in turn supports reading engagement, which in turn further builds comprehension skills and background knowledge (Guthrie et al., 2004; Snow, 2010). It has also been claimed that there may be other moderator variables, such as the design of interventions and participant characteristics that moderate the instructional effects on students’ levels of self-efficacy (Chen, 2022). Inconclusive findings support the postulation that, despite the malleability of students’ self-efficacy, it may take time for students to demonstrate a significant change in their level of self-efficacy. Therefore, more data are needed in order to determine the materials, the conditions, and the processes through which students’ academic self-efficacy can be improved after applying an SRL training program.

### 1.3. The Present Study

A recent report regarding several countries stated that only 7% of students can comprehend lengthy texts, establish distinctions between fact and opinion, based on cues pertaining to the content or source of the information, and deal with concepts that are abstract or counterintuitive (OECD, 2023). The present study sought to add further evidence to the relatively limited literature on enhancing students’ SRL skills with a focus on RC in regular elementary school classrooms, as most of the SRL interventions with a focus on RC have been applied with older students or with reading-disabled students (DeCorte et al., 2001; Lau, 2020). Students in the upper elementary grades must become strategic comprehenders of increasingly sophisticated texts. “They must build a vocabulary of words and concepts as well as a vocabulary of cognitive and metacognitive approaches to texts” (Slavin et al., 2009, p. 1426). However, the large majority of fifth graders are not used, for instance, to reflecting on their own reading process and strategy-oriented instruction is claimed to be difficult to integrate into the reading routines of the students (Souvinger & Mokhlesgerami, 2006). Therefore, an objective of this study was to deliver in the classroom a short-structured, curriculum-based, and relatively simple to apply training course for students that could be employed in the future by the teachers themselves. The main aim of the study was to develop, implement, and evaluate the effectiveness of a training program for upper elementary school students in order to improve their SRL skills in RC, subsequent performance, and reading self-efficacy. The broader context of the training program was thegreek language course. A benefit of the study could be the instructional material produced and the structured practices in order to better equip teachers to strengthen strategic reading and RC in the classroom.

The theoretical background of the intervention was Zimmerman’s (2000) three-phase social–cognitive model of SRL. At the primary school level, training programs that were developed based on social–cognitive theories on SRL have been found to have positive effects (Dignath & Büttner, 2008).

According to the literature presented above, the hypotheses of the study were formed as follows. After the intervention, the EG students’ performance in RC (Hypothesis 1), their reported use of SRL strategies in RC (Hypothesis 2), and self-efficacy in RC (Hypothesis 3) should be significantly improved in comparison to the students of the CG. Finally, students’ perceptions about the effects of the program will be retained for several weeks after the intervention (Hypothesis 4).

## 2. Materials and Methods

### 2.1. Participants

The participants of the study were a convenient sample of 109 students (boys = 49) from grades 5 and 6 (i.e., 11 and 12 years old) from three elementary state schools of a medium-sized city in Greece. The students were randomly assigned to an experimental (EG, *n* = 54) and a control group (CG, *n* = 55) (Table 1).

### 2.2. Research Design

The research design involved an experimental and a control group who took measures of performance, strategy use, and self-efficacy before and after the intervention. In Table 2 the research design of the study is presented.

### 2.3. Measures

*Performance on Reading Comprehension*. Two performance tests on RC were developed for the purposes of the study, one for the pre-measure and one for the post-measure. The texts included were selected in collaboration with the classes’ teachers and they were similar to the texts included in the language school books. The RC tasks included were also developed in collaboration with the teachers. Each test contained a short narrative text of about 300 words that students had to read carefully and answer or carry out the requested tasks. There were various RC tasks, such as open questions checking the students’ understanding, explaining specified phrases or words in their own words, reading rephrased sentences and decide whether their meaning fit the text (right or wrong), reading a passage and detecting the sentence that did not fit the ideas expressed in the text, etc. The two tests were rated as equivalent in difficulty by the teachers. One rater graded students’ pre- and post-tests while a second rater graded one third of the pre-tests. The mean intra-class coefficient for the RC tasks between the two raters’ scoring was 0.80. A sum score for RC was calculated for each student. The scores the first rater assigned served as the students’ scores in the test.

*Strategies for Reading Comprehension*. The students’ strategies for RC were assessed before and after the intervention program with a self-report scale developed by the author (Dermitzaki & Papakosma, 2020). There were 13 items referring to cognitive and metacognitive strategies employed during the text comprehension procedure: e.g., “While reading a text, I underline key words or key phrases”, “After reading a text, I ask questions to myself to check if I understood well”. Answers were given on a 5-point Likert type scale (1 = No/very rare use to 5 = Always/Almost always). A composite score was calculated by adding the students’ answers to the 13 sentences of the scale and dividing by 13. Cronbach’s alpha for this scale before the intervention was 0.82.

*Self-efficacy for Reading Comprehension*. The students’ reading self-efficacy was assessed with five self-referent items adapted from a previous work of the author (Dermitzaki & Efklides, 2000), e.g., “I expect that over the next weeks I will do well on tasks and tests of reading comprehension”. Answers were given on a 5-point Likert type scale (1 = Completely disagree to 5 = Completely agree). A composite score was calculated by adding the students’ answers to the five sentences of the scale and dividing by five. Cronbach’s alpha for this scale before the intervention was 0.61.

### 2.4. Curricular Structure of the Training Program

As stated before, the SRL framework was adopted for implementing strategy instruction in the classroom. Cognitive, metacognitive, and motivational aspects of self-regulated learning were taken-into-account. Teaching of cognitive strategies for RC (e.g., paragraph shrinking, developing a story map), of metacognitive strategies (e.g., self-monitoring, self-evaluation of understanding), and of motivational strategies (e.g., emotion and concentration regulation) was included. Following Zimmerman’s (2000) three-phase model of SRL, the students were instructed in the three phases with regard to reading, that is, what good readers do before, during, and after reading in order to achieve RC. The texts included in reading activities during the program implementation were mainly narrative–expository texts and newspaper informative articles similar to the texts presented in students’ language school books.

According to Zimmerman’s view of the development of SRL, four distinct teaching stages were defined on how to instruct a learning strategy (Zimmerman, 2000, 2002). Learners have to pass through different levels of self-regulation until they achieve a level of control and regulation of their own learning process independently of others in various conditions. In the present study, we followed the first three steps (stages) for instructing self-regulation reading strategies. The first stage refers to discussing the strategy, modeling the strategy, and memorizing it (observational learning), the second stage refers to students’ guided practice (emulation) without achieving full independence, and the third stage refers to independent and deliberate practice across tasks and settings (self-controlled). The SRL strategies included in the program were instructed via various methods. Explicit teaching, modeling, and reciprocal teaching were among the teaching methods employed. Moreover, experiential activities to bring awareness and activities in pairs, in groups, and whole-class discussions took place during the program implementation. Enhancement in RC, thus, was approached as “…a learning transfer, where quite specific new (strategic) skills were integrated into daily reading routines” (Souvinger & Mokhlesgerami, 2006, p. 69). Knowledge about the strategies instructed, that is, when to use them and why they can be helpful in creating meaningful representations as well as frequent feedback about their strategy use was offered (Snow, 2010; Zimmerman, 2000).

Regarding the greek national curriculum for teaching reading in the 5th and 6th grade of elementary school, among its aims it is stated that students are expected to be able to read fluently and with precision texts of increasing difficulty, to accurately comprehend the text information, to search for and to select information, to analyze and synthesize information, to compare, assess, and make good use of information, to be aware of the purpose of reading and to employ the appropriate reading strategies to reach it, to evaluate the text content, format, and text effectiveness, to distinguish the different formats and genres of texts, to apply their reading abilities to other subjects as well as to their social environment, to develop a positive but also a critical attitude towards reading, to become independent proficient readers, and to enjoy the linguistic diversity in different texts (ΙSB, 2011). However, although the curriculum integrates the modern approaches of teaching reading, such as constructivism and critical literacy, it does not explicitly embrace the teaching of strategic reading and self-regulation skills. Moreover, the actual teaching of reading in the classroom is usually more focused on the development of students’ basic skills for meaning construction, thus, instructing mainly cognitive strategies, such as detecting and organizing the main ideas and reviewing the main points, and less on deep processing and metacognitive strategies (e.g., Dermitzaki & Papakosma, 2020). In the present study, we followed the state curriculum for teaching reading and we enriched the teaching of RC in the EG with the principles and skills of SRL described in the following paragraphs.

The training program of the present study consisted of 10 thematic units delivered in 14 regular school teaching hours. During the first two units, the aims of the intervention program, the main concepts, and the group’s expectations were clarified. The students were introduced into the rationale of the intervention, the main concepts (e.g., what “effective” reading is), the factors that can support or impede reading and learning (e.g., personal thoughts and emotions and tips to manage them), and the 3 phases of SRL in RC (before, during, after reading). The example of Odysseus, the hero of Homer’s “Odyssey”, and his adventure with the cyclops Polyphemus was used as an example of successful self-regulated activity. Odysseus conceives a detailed plan of steps in order for him and his comrades to escape from the cyclops’ cave, he implements the plan step by step and monitors its success, and he performs the necessary regulations in action where needed, so that finally he achieves the goal, i.e., to escape. Finally, he reflects back to his lost comrades and the means available and he redesigns the journey back to Ithaca. Accordingly, the students were taught that to improve the possibility of success in text comprehension—and, actually, in any learning activity—the agent should think in advance, plan and organize actions, motivate themself during action to implement the plan and the strategies selected, monitor and regulate activities, and, after action, evaluate the outcomes and reflect.

The next units (units 3 to 7) focused on instruction and training on strategies for self-regulation of reading. The main idea was that, in each phase of the reading processes, i.e., before, during, and after reading, there are some strategies that can work effectively for promoting RC. The example of Odysseus’ execution plan for escaping the cyclops was reminded. For instance, before acting (i.e., reading), orientating the text and planning of the reading process are important (e.g., clarification of the reading goal, preview the text and relevant material, estimating effort and time, tips for self-motivation). Relevant tasks and activities were introduced to the students. During reading, applying effective cognitive and metacognitive strategies was taught in order to comprehend, monitor understanding, and remember ideas and reading material (e.g., generating questions, underlining, concept maps, prediction, summarization, self-monitoring checklists). After reading, self-evaluation of reading attainments was instructed through strategies such as checking understanding and remembering, checking attainment of the initial reading goal, etc.

Units 8 and 9 focused on revising the strategies taught in previous meetings, reminding the phases and steps of the reading process, and extending practice at home to promote students’ independent practice and commitment to continuing using the skills taught for personal improvement in RC. The students were invited to apply the strategies taught during home-studying school texts, such as in history and geography texts, and fill in self-monitoring/self-check forms. Finally, the students of the EG were invited to set their own goal for personal improvement in RC and become committed to continuing using the skills taught. The final 10th unit focused on a general review of the program through a memory quiz and on students’ evaluation of the program regarding usefulness, enjoyment, and suggestions for improvement. An extra closing meeting took place with the EG where students were given a certificate of attendance.

### 2.5. Procedure

The headteachers and teachers of the schools and the parents of the students gave their informed consent for participation in the study. Pre-test measurements took place at the beginning of December. The training program started about one week after the initial measurements, i.e., the first two introductory units were delivered in December whereas the rest of the training units were delivered from January to early March. The training was delivered to the EG by the researcher during regular school language courses after agreement with the class’s teacher (usually during the first half of the school day). The CG received the usual teaching within their school language classes. Post-measurement took place between 8 and 10 days after the end of the intervention. Moreover, three months later, a follow-up study took place.

### 2.6. Follow Up

Three months after the intervention, 12 randomly selected students (boys = 5, 5th grade = 7) of the EG were interviewed with a semi-structured interview in order to investigate the possible gains from the intervention program. The questions included in the interview were: “What do you remember from the program implementation in terms of issues discussed, phases of the reading act, activities, strategies, etc?”, “Have you used in everyday studying of school texts specific techniques or strategies you worked on during the program implementation? What strategies you have used and under what conditions?”, and “What do you think in terms of the usefulness of the strategies taught in order to reach RC?”.

## 3. Results

In order to detect whether there are significant differences before and after the intervention between the EG and the CG in RC performance, in SRL strategy use, and in self-efficacy in RC, a 2 (Time) X 2 (Group) repeated measures MANOVA with time (pre-post) as the repeated measure factor and group (experimental–control) as the between-subjects factor was performed on the data. Performance in RC, SRL strategies in RC, and self-efficacy in RC were the dependent variables. The means and standard deviations of the variables for the two groups appear in Table 3. The multivariate tests showed a significant overall multivariate interaction of Time X Group, *Wilks L* = 0.831, *F*_(3, 97)_ = 6.575, *p* = 0.000, η^2^ = 0.169. The univariate tests showed that the Time X Group interaction was significant for performance in RC (*F*_(1, 99)_ = 6.306, *p* = 0.014, η^2^ = 0.060) and for strategy use (*F*_(1, 99)_ = 14.472, *p* = 0.000, η^2^ = 0.128). The univariate test was not significant for self-efficacy in RC (*F*_(1, 99)_ = 0.036, *p* > 0.05).

Next, separate paired sample *t*-tests for the EG and for the CG were conducted in order to determine whether there was a significant mean difference between paired scores of each group (EG, CG) before and after the intervention. Regarding students’ performance in RC, results showed that it significantly increased after the intervention both for the students of the EG (*t*_(50)_ = −6.814, *p* = 0.000) and for the students of the CG (*t*_(52)_ = −4.216, *p* = 0.000). Regarding the reported strategy use, the results showed that it significantly increased after the intervention for the students of the EG (*t*_(50)_ = −2.243, *p* = 0.029) while, for the students of the CG, the reported strategy use significantly decreased after the intervention (*t*_(49)_ = 3.427, *p* = 0.001).

As both the EG and the CG significantly improved their performance in RC after the intervention, the next step was to investigate whether the degree of improvement in performance scores between the EG and the CG differed significantly. A new dependent variable was calculated by subtracting students’ performance before the intervention from their performance after the intervention. The independent sample *t*-test showed that there was a significant difference in the improvement of performance between the two groups (*t*_(102)_ = 2.364, *p* = 0.020). Specifically, the EG presented significantly larger improvement in comparison to the CG. The mean difference for performance improvement for the EG was *M* = 2.86 (*SD* = 3.00) and for the CG it was *M* = 1.54 (*SD* = 2.67).

### Follow Up

Three months later, 12 students of the EG were interviewed with three open-ended questions. The first question focused on what each student remembered in terms of the issues discussed, concepts, tasks, and activities in order to evaluate their spontaneous recall regarding the content, the concepts, and the activities taught during the program’s meetings. Ten out of twelve students referred to the three phases of SRL (actions I can do before, during, and after reading), seven out of twelve students spontaneously recalled and described at least one specific strategy or technique for RC instructed during the program, such as ways for focusing attention during studying and self-evaluation activities, and six out of twelve students referred to their capabilities for personal responsibility and activation for understanding and learning.

The second question assessed students’ perceptions regarding the usefulness of the program. All the interviewed students considered that the program was very or quite useful and helpful. Seven out of twelve students reported that, after the intervention, their text understanding became easier and deeper, and five out of twelve students thought that their time management and related performance had been improved since the end of the program when studying text.

Regarding the third question on whether the students still use specific techniques or strategies instructed during the program, the most frequently reported strategies were: underlining the important words or sentences (eight students out of twelve), ways to manage intense emotions (eight students out of twelve), skimming the text to orientate and plan the reading process (seven students out of twelve), self-monitoring while reading (six students out of twelve), generating subheadings for each unit/paragraph (six students out of twelve), producing a conceptual plan of the text (five students out of twelve), and self-evaluation/checking memory after reading (five students out of twelve).

## 4. Discussion

It has been postulated that fostering typical elementary school students’ self-regulated learning and related skills with a focus on text comprehension might be beneficial to their respective attainments and strategy use, as evidence attests that fully competent reading is not common in elementary students (Dignath & Büttner, 2008; Pressley & Gaskins, 2006; Schünemann et al., 2013; Souvinger & Mokhlesgerami, 2006; Thiede & de Bruin, 2018). The present study sought to add further evidence to the relatively limited literature on this topic, aiming at improving elementary students’ performance in RC, SRL skills in RC, and reading self-efficacy. Overall, the results of the study showed that implementing the present short-structured strategy instruction program in upper elementary school students, based on an SRL theoretical framework, significantly improved the students’ performance in RC as well as their reported strategy use in RC. No differences in students’ reading self-efficacy were detected after training.

More specifically, the first hypothesis of the study stated that, after the intervention, the EG students’ performance in RC should be significantly improved in comparison to the students of the CG. The analyses of the study showed that both the EG and the CG significantly improved their performance in RC after training. An overall improvement in knowledge, skills, and consequent performance in all students would be expected, as not only did the EG receive training through the intervention program but the CG also received the usual instruction during regular school language classes. However, the EG presented significantly larger improvement in their RC performance in comparison to the improved performance of the CG. We might argue that this larger performance improvement of the EG in comparison to the CG reflects the positive effects of the intervention where participant students were trained in self-regulation strategies before, during, and after reading within their classroom context. The additional finding related to Hypothesis 2 that only the EG’s reported SRL strategies in RC were significantly improved after the intervention, and not the CG’s, strengthens the above conclusion. Hence, the study adds further support to past studies with elementary school students showing positive effects on RC when employing SRL training methods (Schünemann et al., 2013; Souvinger & Mokhlesgerami, 2006; Stoeger et al., 2014).

Furthermore, Hypothesis 2 stated that, after the intervention, the EG students’ reported use of SRL strategies in RC should be significantly increased in comparison to the students of the CG. This hypothesis was confirmed as it was shown that only the EG’s reported SRL strategies in RC were significantly improved after the intervention. As stated before, the training program focused on educating students about using different SRL skills and strategies (cognitive, metacognitive, motivational), that is, strategies to organize and elaborate text material and to control and regulate the process of understanding in order to achieve text comprehension. Thus, this finding is in line with other studies reporting that after direct strategy training a significant improvement was observed in terms of students’ application of reading strategies (DeCorte et al., 2001; Schünemann et al., 2013; Souvinger & Mokhlesgerami, 2006; Stoeger et al., 2014). It was also found that the CG’s reported strategy use significantly decreased in the post-test phase. This might be due to the fact that the CG, by completing the scales about strategy use in RC, became aware of the need to be strategic, in combination with the fact that they did not receive related systematic training. The present study could be replicated in the future using assessment of students’ actual skills as they unfold during reading and not only their reports. Such findings would further enlighten this area and they would be of great value to researchers and educators.

The above results should be interpreted with reference to the specific educational context investigated. In the greek national curriculum of teaching reading, the modern approaches of teaching reading are taken-into-account (e.g., constructivism, critical literacy). Therefore, both groups (EG and CG) improved their RC performance after training. However, the EG presented significantly larger improvement of their RC performance in comparison to the improved performance of the CG. In our view, further refinements of the curriculum can be made both in terms of its aims and rationale and in terms of suggested practices and teaching methods. For example, the teaching of strategic reading could be more visibly and strongly embraced in the curriculum and teachers could be supported and further educated on how to foster strategic reading via organized and targeted instructional steps. In terms of the actual teaching of reading in school classrooms, evidence supports that it is frequently more focused on the development of students’ basic skills for meaning construction and less on deep processing and on metacognitive strategies (e.g., Dermitzaki & Papakosma, 2020). The present study offers evidence on the benefits of a short-structured instructional program on RC for a typical population both to students’ reported strategy use and to their RC performance.

Thus, it seems that, within the SRL framework, students’ planned and active engagement in implementation of different SRL skills and strategies (cognitive, metacognitive, motivational) during regular classes might be an effective means of improving their RC performance and their respective skills as reflected in students’ reports. For achieving comprehension, the reading process involves conscious effort and the use of appropriate strategies on behalf of the reader would be valuable to ensure that the process of reading is focused on building mental representations for meaning construction and not just on reading the material (Cromley & Azevedo, 2007; Kintsch, 2009; Snow, 2010). In future studies, the influence of other mediators on reaching RC and the sustainability of training effects of SRL on RC, such as students’ metacognitive knowledge about reading, should also be studied, as pointed out by researchers (Spörer & Schünemann, 2014).

Regarding Hypothesis 3, it was predicted that, after training, the EG students’ self-efficacy in RC should be significantly increased in comparison to the CG students. This hypothesis was not confirmed as both groups’ self-efficacy in RC remained at the same levels at pre- and post-intervention. This finding does not align with studies reporting positive, although relatively weak, effects of training programs on students’ self-efficacy in reading and learning (Chen, 2022; Panadero et al., 2017). Rather, it is congruent with findings that did not report significant differences in students’ self-efficacy after SRL skills training, regarding both typical students and students with reading difficulties or disabilities (Cerezo et al., 2019; Cosentino, 2017; Cleary et al., 2017; Nelson & Manset-Williamson, 2006; Souvinger & Mokhlesgerami, 2006). Hence, regarding the observed stability of the reading self-efficacy in our study, one explanation could be that students do not immediately automatize the improved strategy use in everyday reading and they might have difficulties overcoming possible feelings of uncertainty in order to change their beliefs to more positive ones (Souvinger & Mokhlesgerami, 2006). The limited hours of training, i.e., the short-term intervention of this study, might be related to this finding. The 14 teaching hours hardly provide sufficient time to internalize improvements in skills and performance and to establish automatized strategy use. Therefore, we encouraged students to implement the rationale and the strategies taught in other subjects as well, such as in history and geography, both during training and after the end of it. Thus, it is not easy to change students’ self-efficacy expectations within a short training period, and it takes time for students to demonstrate a significant change in their level of self-efficacy (Dignath et al., 2008). The total amount of instruction time, the duration of each session, and frequency of sessions might positively moderate the instructional effects on students’ levels of self-efficacy (Chen, 2022). In addition, another explanation for the rejection of Hypothesis 3 could lie in the complex relations between using SRL strategies and academic self-efficacy shown in some studies. The relationship between strategy use and self-efficacy may be mediated by the different characteristics that learners bring to the classroom (Chen, 2022; Graham et al., 2020). For instance, indirect effects of training on self-efficacy through improving students’ knowledge of SRL strategies have been reported (Cerezo et al., 2019). Thus, it seems that the evidence on the impact of SRL comprehension strategy instruction on students’ self-efficacy from studies with participants either with or without learning or reading disabilities is not conclusive. Future research should define the individual and contextual factors that might be associated with this relationship.

Finally, it was hypothesized that students’ perceptions about the effects of the program will be retained for several weeks after the intervention (Hypothesis 4). The interview data revealed that, three months after the intervention, a good number of the students being interviewed recalled spontaneously the phases of SRL and specific strategies that had been instructed, but they also conveyed the general rationale of the intervention, that is, the reader’s personal responsibility and active engagement during reading. Moreover, a significant number of students reported that they are still using specific techniques or strategies instructed during the program, including cognitive (such as underlining the important words or sentences, skimming the text before reading, generating subheadings for each unit/paragraph), metacognitive (such as self-monitoring, self-evaluation/checking memory after reading), and motivational–affective (i.e., emotion management). Finally, regarding students’ perceptions about the usefulness of the program, all the interviewed students considered that the program was very or quite useful and helpful and many of them have noticed in themselves signs of improvement in terms of text understanding, time management, and related performance since the end of the program. Thus, students’ active engagement in SRL skills during everyday reading activities seems to be effective to prevent the frequently observed decline in the use of newly acquired strategic skills over prolonged follow-up intervals. Therefore, one could argue that Hypothesis 4 of the study has been confirmed. In the future, a later follow-up study, e.g., several months later, could be added in order to test for long-term effects of the training program. Moreover, such a study should include more direct assessments of students’ use of reading strategies, via questions on specific strategies or via recording the actual use of students’ strategies (Vettori et al., 2023).

All in all, the present study showed that elementary students’ reported strategies in RC and respective performance can be significantly improved when students become actively involved in a structured program of SRL skills training embedded within their regular school language courses. The current study is innovative in that a specific model of SRL is embedded within typical students’ classes and students learn about it and then work systematically through the specified steps and skills of the model. Thus, reading strategies instruction using an SRL framework can be successfully implemented in a whole-classroom setting. Instructional process programs designed to change daily teaching practices regarding reading have obtained substantial research support (Slavin et al., 2009; Trabasso & Bouchard, 2002). The gains of such interventions might be maintained for several weeks after the end of the training program, at least as regards students’ perceptions and representations, as the present study revealed. In order to maximize the gains, we should aim at increasing students’ self-efficacy for RC that is significantly associated with students’ further SRL practices, perhaps through longer-term training.

The findings and conclusions of the present study must be taken-into-account with caution due to its limitations. The small number of participant students in this study does not allow generalization of the present findings to the upper elementary school population. Further evidence is needed using larger numbers of participants from various schools with diverse backgrounds. Moreover, the training program, although effective in SRL strategy use and in RC performance, did not meet the goal of improving students’ reading self-efficacy. Therefore, a longer-lasting and perhaps more intense training program for elementary school students might be designed and embedded within language classes. Further, in this study, the use of SRL strategies was assessed via students’ self-reports and not through direct measures of actual strategy use. Self-report measures of SRL should be used with caution when examining SRL skills in classroom contexts (Dermitzaki et al., 2009; DiFrancesca et al., 2016). Finally, the specific kind of texts used and the level of their complexity both in training and in pre- and post-tests restrict general conclusions regarding the field of RC (Rollins et al., 2022; Snow, 2010). RC is a complex skill and important cultural, educational, and individual differences in the conceptualization of text comprehension exist. To provide students with skills to deal with all kinds of texts on their own is an optimal goal of education (Souvinger & Mokhlesgerami, 2006).

The results of this study corroborate that it might be beneficial to implement strategy-oriented reading and self-regulation skills for all students independent of their skills as early as possible (Souvinger & Mokhlesgerami, 2006). Such findings suggest that a focus on language learner strategies, within an overarching self-regulatory framework, is of value to researchers and it is also of value to practitioners, especially in contexts characterized by heterogeneous learner populations (Graham et al., 2020; Perry et al., 2007). It is underlined that when students have opportunities to become aware of their learning processing, they have greater opportunities to adapt (Winne, 2018). Accordingly, teachers could offer opportunities for students to become aware of their reading processing, to monitor and observe how they read and construct understanding, and to allow time for experimenting with text comprehension strategies and processes. Personal processes, the environment, and individual behaviors of both teachers and students are factors that facilitate students’ use of SRL strategies in reading. The findings of this study add to a body of evidence to the effect that what matters for student achievement are approaches that aim at improving what teachers and students do together every day. However, evidence attests that strategy-oriented instruction seems unfamiliar to teachers and that teachers frequently do not think that SRL is teachable, therefore, they usually do not explicitly teach strategies that promote self-regulated learning and reading in students (Lawson et al., 2019; Souvinger & Mokhlesgerami, 2006; Spruce & Bol, 2015). To adequately embed the teaching of self-regulation reading skills in authentic classroom environments there is a need to educate teachers and students in classroom strategies by fostering their knowledge and practices for SRL implementation and by providing teachers with evidence-based material to maximize students’ participation and engagement in self-regulation of RC. Further evidence from longitudinal implementation of the present training program in a variety of group and situational conditions is needed in the future. Predicting success in comprehension requires taking into account factors related to the reader, to the text being read, to the task being undertaken, and to the sociocultural context in which the reading is occurring (Snow, 2010).

## Figures and Tables

**Table 1:** Participants of the study.

	Experimental Group (*n* = 54)		Control Group (*n* = 55)		
	Boys	Girls	Boys	Girls	Total
Grade 5	14	24	9	10	57
Grade 6	11	5	15	21	52
Total	25	29	24	31	109

**Table 2:** The research design of the study.

Phase	Experimental Group (*n* = 54)	Control Group (*n* = 55)
Measures before the intervention	-Self-efficacy in reading comprehension -SRL skills for reading comprehension -Performance in reading comprehension	
Intervention	14 h of instruction on RC skills	Regular teaching of RC within language course
Measures after the intervention	-Self-efficacy in reading comprehension -SRL skills for reading comprehension -Performance in reading comprehension	
Follow-up	Retrospective interviews with 12 students of the EG	

**Table 3:** Means and standard deviations of the variables for the two groups.

	Experimental Group (*n* = 54)		Control Group (*n* = 55)		
	M	SD	M	SD	*p*
RC performance pre-test	9.84	2.95	10.24	2.86	
RC performance post-test	12.70	2.76	11.68	2.49	0.014
SRL strategies pre-test	3.14	0.79	3.03	0.63	
SRL strategies post-test	3.38	0.56	2.77	0.68	0.000
Self-efficacy in RC pre-test	4.00	0.44	3.87	0.47	
Self-efficacy in RC post-test	4.05	0.52	3.88	0.48	ns

Note: “RC”: Reading Comprehension, “SRL”: Self-Regulated Learning.

## Data Availability

The raw data supporting the conclusions of this article will be made available by the author without undue reservation.
